# Current trends in biomarker discovery and analysis tools for traumatic brain injury

**DOI:** 10.1186/s13036-019-0145-8

**Published:** 2019-02-19

**Authors:** Briana I. Martinez, Sarah E. Stabenfeldt

**Affiliations:** 1 School of Life Sciences, Arizona State University, Tempe, AZ USA; 2 School of Biological and Health Systems Engineering, Ira A. Fulton School of Engineering, Arizona State University, PO Box 879709, Tempe, AZ 85287-9709 USA

**Keywords:** Traumatic brain injury, Biomarkers, Phage display, Omics, Imaging, Machine learning

## Abstract

Traumatic brain injury (TBI) affects 1.7 million people in the United States each year, causing lifelong functional deficits in cognition and behavior. The complex pathophysiology of neural injury is a primary barrier to developing sensitive and specific diagnostic tools, which consequentially has a detrimental effect on treatment regimens. Biomarkers of other diseases (e.g. cancer) have provided critical insight into disease emergence and progression that lend to developing powerful clinical tools for intervention. Therefore, the biomarker discovery field has recently focused on TBI and made substantial advancements to characterize markers with promise of transforming TBI patient diagnostics and care. This review focuses on these key advances in neural injury biomarkers discovery, including novel approaches spanning from omics-based approaches to imaging and machine learning as well as the evolution of established techniques.

Traumatic brain injury (TBI) affects an estimated 1.7 million people in the United States each year and is the leading cause of death in young adults and children in industrialized countries [1–4]. Individuals with TBI are likely to develop cognitive and sensorimotor impairments, such as decreased processing time, memory loss, and difficulties using fine motor coordination [5–7]. Furthermore, individuals with TBI are more likely to acquire neurodegenerative diseases such as Alzheimer’s Disease (AD) and Parkinson’s Disease (PD) later in their lifetime [8–10]. In the United States alone, the direct (hospital treatments) and indirect (loss of productivity, lost wages) costs of TBI in 2010 were estimated at $76.5 billion [11]. Thus, TBI is of major public and economic concern.

TBI should be viewed as not a single pathophysiological event, but a cascade that involves two separate injury phases (Fig. 1). The initial insult triggers the primary injury process, which results in tissue deformation, necrosis, and shearing of neurons, axons, and glial cells [12]. The mechanical force disrupts the blood-brain barrier (BBB), typically reaching maximum permeability within a few hours of the initial insult [13, 14]. Glutamate released from damaged nerves then trigger a secondary injury cascade, which causes edema, increase of pro-inflammatory cytokines, and ischemia [12, 15]. This secondary cascade persists for weeks to months after the initial insult, causing an accumulation of cell damage and death [16, 17]. This heterogeneous environment varies on a case by case basis dependent upon anatomical site of the injury, injury phenotype (e .g., closed head trauma vs penetrating brain injury), severity, and age of patient at time of injury [18–20].

**Fig. 1:**
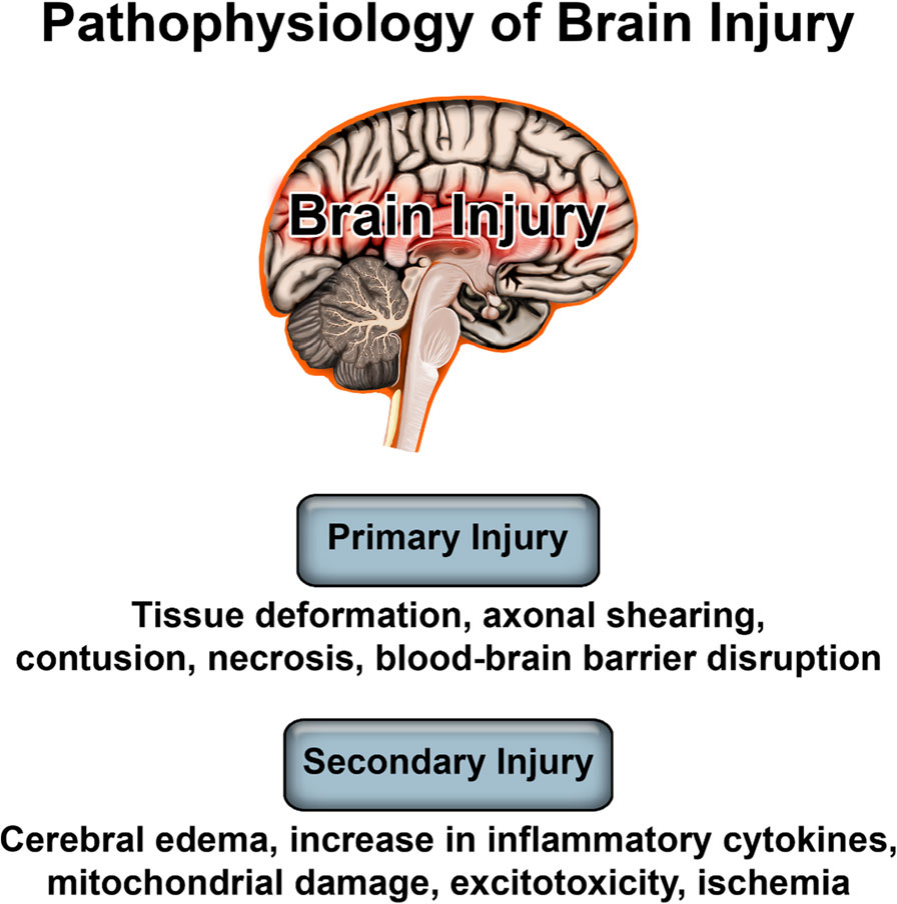
TBI pathophysiology. The primary injury, caused by the initial insult, contributes to a secondary injury progression

Since the complexities of the injury microenvironment are still not fully elucidated, this heterogeneous pathology is a primary barrier to developing sensitive diagnostic tools. The Glasgow Coma Scale (GCS), a commonly used survey in emergency room settings, diagnoses TBI with a battery of observations such as patient’s eye and motor response to stimuli. Despite being a hallmark of TBI diagnosis, the GCS has been found to be a poor predictor of patient outcome and is not appropriate for patients with prior neurological conditions [18, 21, 22]. Similarly, traditional computerized tomography (CT) and magnetic resonance imaging (MRI) scans are reliable for visualizing fractures, hematomas, and edema, but may have difficulty capturing more mild characteristics of brain trauma [18, 23]. Diagnostic inaccuracy is detrimental to patient well-being, as patients who are incorrectly diagnosed may receive sub-optimal treatments as their quality of life decreases.

Researchers are now turning to biomarkers, objective molecular signatures of injury, as a platform for developing more sensitive and specific TBI treatment and diagnosis tools. Identification and quantification of biomarker expression provides the basis for producing these technologies. For example, a biosensor targeting TBI biomarkers can potentially work to both diagnose TBI patients and monitor the severity of their disease progression. Further, these tools may provide insight on treatment efficiency by assessing changes in biomarker expression. Several biomarkers for TBI have been identified, mostly located in serum or cerebral spinal fluid after injury, including indicators of inflammation, necrosis, apoptosis, and astrocytosis [18, 24]. There have been several clinical trials analyzing the reliability of using biomarker expression as an indicator of disease progression [25–27]. While various biomarkers of injury have been identified, such as glial fibrillary acidic protein (GFAP), S100beta, and ubiquitin carboxyl-terminal hydrolase isozyme L1 (UCH-L1), the utility as TBI diagnostic markers in the clinic is debated due to lack of specificity and sensitivity to TBI [28, 29]. These confounding results may be attributed to several factors of polytrauma, including time post-injury, severity, and injury phenotype.

Due to the complex heterogeneity of TBI, biomarker discovery in preclinical models must consider the limitations of each model when characterizing candidate biomarkers. Although no one animal model can recapitulate the full complexity of TBI, they have distinct characteristics that can aid researchers in discovery of biomarkers associated with different aspects of TBI pathology. Focal injury models, such as the controlled cortical impact (CCI) model, produce cavitation, contusion, vasogenic and cytotoxic edema [12, 30]. While focal injury models are clinically relevant to edema in TBI patients, diffuse models share characteristics with TBI experienced by athletes and military personnel [12, 31]. Factors such as high intracranial pressure and progressive gray matter degradation are investigated are often investigated using diffuse injury models, such as the fluid percussion injury (FPI) [12]. Blast-induced injury models in particular are designed to reflect TBI in military conflicts by using compression shock tubes to induce blast waves [12, 32]. This model produces an array of symptoms highly relevant to human blast-induced TBI, such as axonal injury, diffuse edema, and prolonged behavioral deficits [32, 33]. Another subset of models known for their human relevance are weight-drop models. These injuries are produced by a free-falling weight onto an intact or non-intact skull and specifically mimics the biomechanics of human TBI induced by falls or vehicle accidents [34]. This technique produces a mix of focal and diffuse injury dependent on the model, and results in neural inflammation, contusion, and hemorrhage [35]. Biomarkers developed with these models can provide unprecedented insight for injury mechanisms and have potential to translate for prognostic and therapeutic use in the clinic.

Currently, there are no approved TBI biomarkers for clinical treatment or diagnostic purposes [18]. Biomarker discovery is an ongoing subfield of TBI research due to the critical need of biomarkers for development of clinical tools. Currently novel biomarker discovery methods are emerging to detect markers that may be further characterized and validated for their translational utility, with each approach having distinct advantages and disadvantages (Table 1). This review will focus on current trends in biomarker discovery tools for TBI, including innovations on established techniques and novel approaches to elucidating the neural injury environment.

**Table 1:** Advantages and disadvantages of biomarker discovery approaches

Discovery Approach	Advantages	Disadvantages
MicroRNA transcriptomics	miRNAs are more abundant in human biofluids than proteins, making them more accessible as biomarkers [43]	miRNA expression may vary due to specific conditions such as fasting, introducing variability in analysis [43]
Neuroproteomics	Elucidate signal transduction events associated with biochemical processes of injury [63]	Large datasets require sophisticated bioinformatics software [17]
Metabolomics/Lipidomics	Metabolites proximity to CSF and brain and ease of lipid transport make them easily detectable [73, 79]	Subject’s environment affects metabolome, possibly producing unwanted variation in data [74]
Phage display	Screening can directly take advantage of heterogeneous injury environment [100]	Requires high throughput sequencing to prevent selection of false positives [104]
Diffusion tensor imaging	Sensitive to detection of diffuse axonal injury and white matter microstructure [111]	Prone to partial volume effect, which may produce false positives [125]
Single-photon emission computed tomography	More sensitivity than CT for detecting lesions, capable of detecting cerebral blood flow abnormalities [109, 131]	Less specificity detecting in vivo morphology [131]
Machine learning	Uncovers nonlinear and higher order effects of predictive variables to model complex relationships [149, 137]	High volume of data required for accurate prediction [148]

## omics-based approaches

### MicroRNA transcriptomics

MicroRNAs (miRNAs) are single-stranded RNAs of 17–25 nucleotides in length and are responsible for regulating gene expression at the post-transcriptional level [36]. These miRNAs can be collected from either tissue or serum, and are screened using either deep sequencing or microarray methodologies. This technique is already emerging as a means for elucidating mechanisms of other central nervous system (CNS) disorders, such as AD, PD, and stroke [37–40], demonstrating its sensitivity with complex neural environments and showing promise as a possible avenue for TBI biomarker discovery. By analyzing miRNA expression in distinct neuropathologies, researchers are able to identify significant changes in gene expression profiles that may contribute to distinct mechanisms of injury, such as temporal injury progression and injury severity [41, 42]. Due to their early expression, miRNAs could be potentially used in point-of-care applications to inform clinicians of the severity of a patient’s trauma [43]. Currently, companies are exploring surface plasma resonance and nanoparticle-based approaches to increase detection of miRNAs to develop sensitive point-of-care technology [43–46].

Biomarker discovery through miRNA expression also has immense clinical utility due to the non-invasive nature of analyzing gene expression through plasma samples and ease of analysis due to advances in microarray and high throughput sequencing technology. Studies utilizing this approach have demonstrated the ability to discriminate TBI patients from non-injured controls. A 2018 study conducted by Qin et al. exhibited this capability by identifying miR-319 and miR-328-5p as miRNAs indicative of severe TBI in comparison to mild or moderate TBI in patients [47]. Similarly, Yang et al. found that specific miRNAs identified in previous microarray studies, miR-93, miR-191, and miR-499 had significantly increased expression in patients with severe TBI and poor prognosis [48–50].

Screening for modulated miRNAs in saliva samples is an approach that has demonstrated powerful detection sensitivity while maintaining the non-invasiveness that makes miRNA analysis so beneficial to research in patient populations. In a 2017 case study, Hicks et al. found that 6 specific miRNAs in the saliva of children with TBI were significantly modulated from control samples, with three of those miRNAs associated with neuronal development [51]. Further, they identified miR-320 as a miRNA directly correlated with reports of attention dysfunction [51], showing utility in providing critically needed age-appropriate biomarkers of injury [52, 53]. Samples taken from concussed athletes also revealed five miRNAs that were significantly upregulated in comparison to non-injured sample expression [54]. When screening for inflammatory proteins in those same samples, analysis revealed no significant difference between groups, suggesting that miRNA analysis may have more sensitivity to certain aspects of the neural injury microenvironment. While promising, it is important to note that miRNA analysis of saliva is relatively new to biomarker discovery literature, and more in-depth research must be done to further test its sensitivity in the clinic.

miRNA expression methods have also shown the same promise in identifying markers of severity as studies conducted in animal models. Balankathiresan at al. found that a blast-induced injury model produced five serum miRNAs were significantly altered in injury groups when compared to control animals at three distinct injury timepoints [55]. Similarly, microarray analysis conducted by Lei et al. revealed hundreds of significantly modulated miRNAs at 6, 24, 48, and 72 h post injury in rat model of diffuse injury [49]. Several miRNA array studies have revealed similar results, with various injury timepoints yielding tens to hundreds of differentially expressed miRNAs in comparison to sham controls using multiple different injury models [56–58]. Further, microarray analyses have revealed miRNAs to reveal essential information about key cellular pathologies in the injury process. For example, miRNA-21, identified by Redell et al. [56] as an indicator of neural injury, has been characterized as a marker indicative of injury progression in aged brains. Sandhir et al. found that miRNA-21 expression increased significantly in injured adult (5–6 months) mice but decreased in aged (22–24 months) mice [59]. However, this decreased expression lead to an upregulation in miRNA-21 targets such as PTEN and RECK, consequently increasing the probability of poor prognosis [59]. From these findings, we can expect for miRNA array analysis to be tremendously beneficial to not only identifying biomarkers of injury, but biomarkers of distinct temporal injury events that may go undetected otherwise. Similarly, biomarkers of injury severity can also be characterized by analyzing miRNA expression. When using a weight-drop model of mTBI with four varying severities, Sharma et al. found that injured animals had a significant increase in miRNAs in comparison to sham controls, while seeing a steady increase in the number of modulated miRNAs correlating to injury severity [60]. These findings were corroborated by a 2017 study that used the same model and severity scale, but also identified the modulated miRNA’s targets, such as calcium signaling pathways [61].

### Neuroproteomics

Neuroproteomics, the study of protein complements of the genome, seeks to analyze protein expression within the CNS to answer questions about disease states and progression [62]. Recently, neuroproteomics approaches have been applied to neurotrauma to identify possible protein biomarkers of TBI, a logical step considering the surge of success with the search for genomic biomarkers [62]. In contrast to genomics analysis, neuroproteomics can elucidate signal transduction events associated with biochemical processes of injury [63]. First, the protein complex is fractionated either by electrophoresis or chromatography. Then, the fractionated proteins are identified and quantified by mass spectrometry. Advances in mass spectrometry have provided researchers with the capability to collect an immense amount of data from proteomes, giving an in-depth look at the global protein environment [62, 64, 65]. Due to the substantial volume of data gathered, neuroproteomics is often coupled with bioinformatics and systems biology to identify proteins of interest and analyze their interactions with other proteins to specific pathways associated with the target condition. .

The specificity and sensitivity of neuroproteomics approaches have been successfully demonstrated with animal models of TBI. Boutte et al. used this technique to assess protein expression in cerebral spinal fluid (CSF) and brain tissue within the acute timepoints of a penetrating ballistic-like brain injury (PBBI) rodent model of TBI. In addition to observing significant expression changes of UCH-L1, this method was able to isolate cullin 1, protein phosphotase 2C-alpha, and minichromosome maintenance protein 2 homolog, proteins associated with neurite outgrowth and cell differentiation, as potential candidate biomarkers of injury, demonstrating the power of utilizing bottom-up discovery techniques with advanced proteomic methodology [66]. A similar study found collapsin response mediator protein-2, dehydrogenase, and synaptotagmin were significantly expressed in cortical tissue samples of rats with focal injury when compared to naïve samples [67]. Using a similar injury model, a study by Thelin et al. found several proteins differentially expressed in correlation with temporal stages of injury. For example, aldolase C showed increased expression at earlier timepoints after injury while hypoxia inducing factor -1a and amyloid precursor protein showed increased expression 2–4 weeks post-injury [68]. Other studies assessing the temporal profile of injury have been conducted, revealing several candidate markers that may be influenced by temporal mechanisms of the microinjury environment [69, 70]. While not yet heavily researched, neuroproteomics may also have utility in the clinic due to the relative ease of analyze whole proteomes of biofluid samples. From the CSF and blood of injured patients, Halford et al. analysis revealed candidate astroglial markers of injury such as aldolase C and astrocytic phosphoprotein [71]. Overall, neuroproteomics takes advantage of the advances in data output and cost of proteome analysis to adequately discover novel candidate biomarkers.

### Metabolomics and Lipidomics

An alternative to neuroproteomics is metabolomics, the study of global metabolic profiles in specific conditions and diseases using mass spectrometry or nuclear magnetic resonance spectrometry [72, 73]. This technique is beneficial for biomarker discovery due to the disruption of homeostasis after injury that is reflected in the metabolome [74]. Similar to neuroproteomics, applying a metabolomics perspective when exploring the injury microenvironment can give rise to novel biomarker candidates not well discussed in the literature. For example, analyzing plasma metabolomics of rats with focal injury revealed significant differentially expressed galactose, demonstrating its capability as an early marker of acute TBI [75]. Several studies have used metabolomics in animal models of TBI to report similar findings of novel candidate biomarkers, including adenosine diphosphate (ADP) and spermidine [76, 77].

Lipidomics, a subset of metabolomics, is emerging as a new approach to biomarker discovery in TBI. The rationale for using lipidomics over neuroproteomics is that lipid expression in blood is reflective of expression in brain tissue and therefore has more clinical utility [78, 79]. Further, CNS tissue has the highest lipid content of any tissue type excluding adipose tissue, and also has a high diversity of different sub-types of lipids [80, 81]. This relatively new approach to injury biomarker research is already demonstrating diagnostic capability in rodent models of TBI. Analyses on the serum lipidome of rodents with a CCI revealed that polyunsaturated fatty acids and sphingolipids are significantly upregulated after injury and may serve purpose as a quantifiable TBI biomarkers [79, 82]. In the other direction, analyzing the plasma of injured mice revealed significant decrease of ether phosphatidylethanolamine levels 3 months post-injury in comparison to controls [83]. Utilizing lipidomics approaches to study perioxidative processes of lipids is also informative of possible biomarkers associated with injury-induced oxidation. For example, Bayir et al.’s analysis of rat cortical tissues after focal injury revealed cardiolipin, a mitochondria-specific phospholipid, may be indicative of apoptosis and oxidative stress [84]. A similar study conducted with the same rodent model of injury found increased levels of 8-epi-prostaglandin F_2α_, a marker of oxidative damage, at 6 and 24 h post-injury [85]. Despite overwhelming evidence of the possible utility of applying lipidomics to biomarker discovery research, very few studies with human patients exist in the literature at this time. However, these studies have shown promise in positively identifying lipids that may be associated with TBI and its neuropsychological outcomes, such as posttraumatic stress disorder [86].

## Phage-facilitated discovery

**Fig. 2:**
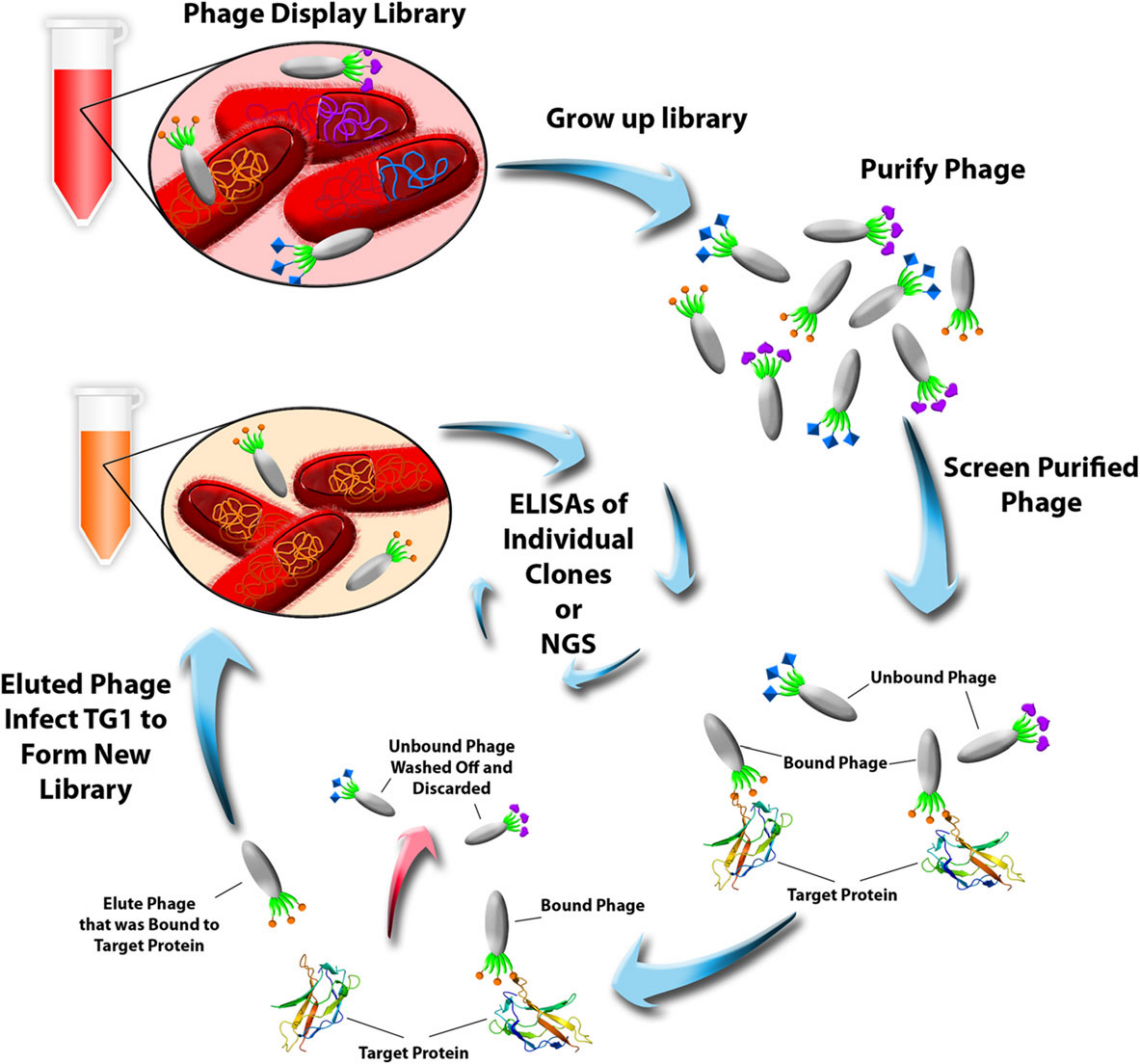
Phage display biopanning process. Phage libraries are grown and incubated with target antigens. Bound phage are rescued and amplified to generate a new library, which is used in subsequent biopanning rounds. Generally, phage selected through this process are validated for specificity with sequencing and ELISAs

Phage display is a powerful screening/selection process that is often utilized in drug discovery research [87, 88]. First described in 1985, phage display has the capabilities of elucidating biological mechanisms by revealing protein-protein interactions [89–91]. Briefly, George P. Smith’s 1985 work provides the foundation for modern phage display technology, in which biological motifs (e.g. peptides, DNA, or antibody fragments) are fused to the gene III of filamentous bacteriophage, such as M13 phage. This fusion results in the bacteriophage “displaying” the motif on its surface with the specific sequence encoded in the gene’s DNA [89]. Large libraries (diversity of 10^6^–10^11^ different ligands) of biological motif-displaying bacteriophages can then be generated to screen against a target antigen or tissue. Collecting only target bound bacteriophage followed by subsequent amplification in bacterial hosts creates a new phage library that is biased toward the target antigen or tissue, thereby completing a single screening cycle, also known as “biopanning”. Biopanning is repeated several times to enrich for biological motifs that have strong affinity for the target antigen or tissue. Upon completion of biopanning rounds, bacteriophage plasmids are sequenced and analyzed for discovering biological motifs that may bind specifically to the target (Fig. 2) [92]. This technology has been used in many pathologies to discover novel biomarkers, for example ovarian cancer and atherosclerosis [93, 94].

Ghoshal et al. explored the feasibility of using phage display as a biomarker discovery tool for TBI using the serum of a focal injury model as target for biopanning [95]. Proteomic analysis (protein pull-down and mass spectrometry) of the converged peptide sequence revealed GFAP as the target antigen. Similarly, phage biopanning can be applied to in vivo screening applications. Phage display has the advantage of being able to target brain vasculature under normal BBB conditions [96, 97]. Further, phage displayed peptides and antibody fragments have the ability to target or transmigrate across the BBB, which is the primary bottleneck of drug development for neurological conditions [98, 99]. Therefore, using this method on an animal model of TBI in vivo may yield peptides or proteins with high affinity to the neural injury milieu. An additional advantage of in vivo biopanning as opposed to a traditional in vitro or ex vivo screening methods is that the former embraces the heterogeneous injury pathology as it unfolds in the neural milieu, creating an opportunity for increased biomarker discovery of TBI. Mann et al. capitalized on this concept and performed in vivo screening in a rodent model of focal TBI [100]. Through this methodology, a novel short peptide, Cys-Ala-Gln-Lys (CAQK), was identified as a unique targeting motif of acute brain injury. To validate specificity, a liver injury model was analyzed and showed no accumulation of the CAQK peptide [100]. The authors exploited this discovery for targeted therapeutics, which demonstrates this method’s feasibility of identifying distinct biomarkers of injury severity and progression.

Despite successful identification of unique ligands of disease and injury in AD and stroke respectively, utilization of phage display for TBI biomarkers has not been thoroughly conducted [101–103]. This slow adoption may be in part due to the difficulty of identifying biomarker candidates from the biopanning process. Traditionally, phage display screening from biopanning involved Sanger sequencing, which only captures genomic analysis of a small fraction of the phage population. The advancement of next generation sequencing (NGS) has improved this process, increasing the sequencing space from 100 clones to 10^7^ clones and consequentially uncovering more positive biomarker candidates for further validation [104, 105]. Additionally, NGS analysis specifically for phage libraries has evolved to development of user-friendly programs [106–108]. Overall, the combination of phage display and NGS for TBI biomarker discovery is promising yet requires more validation to fully achieve its potential.

## Imaging

Due to its application in hospital settings, especially within the first 48 h of injury, researchers have made strides in discovering biosignatures that are detectable by imaging modalities. These imaging-based diagnostic approaches inform clinicians on trauma severity and can also aid in evaluating the progression of injury with routine monitoring. Despite its common use and great capability of visualizing severe trauma, such as skull fracture, hematoma, and edema, traditional imaging tests such as CT and MRI may have difficulties detecting subtle aspects of brain injury [109, 110]. Experimental and clinical researchers are now improving sensitivity of these imaging techniques and using them to detect biosignatures of neural injury that are not seen in control populations, leading them to characterize and validate candidate biomarkers of TBI.

Recent efforts have explored the utility in employing diffusion tensor imaging (DTI) as a tool for analyzing possible biomarkers of injury in patients. In contrast to traditional MRI, DTI provides the ability to spatially map white matter and analyze its diffusivity via fractional anisotropy. This feature shows promise in being more sensitive to detection of axonal injury as opposed to traditional MR techniques alone [111], which is significant due to traumatic axonal injury (TAI) being a key contributor to cognitive dysfunction in TBI populations [111, 112]. Animal studies employing DTI as a discovery and validation technique have shown success in analyzing biomarkers of injury. Mac Donald et al. found promising results when using DTI in conjunction with histological analysis compared to common MRI analysis when imaging rodent focal injury model brains. Their analysis suggested that DTI was able to detect significant changes in axial diffusivity and relative anisotropy, validated by amyloid beta precursor protein histology. Meanwhile, MRI of the same region was not able to detect this axonal injury, only the contusion [113]. Several other studies using both rodent and porcine models have corroborated these results [114–116], further demonstrating both the utility of axonal diffusion as a candidate biomarker and DTI as a possible imaging tool for the validation of this biomarker.

Clinical applications of DTI are also being heavily researched with high levels of optimism. Rangaprakash et al. applied DTI in an effort to differentiate chronic mild TBI patients from non-injured controls, and found a significant loss of integrity of white matter fibers in hippocampal-striatum pathway in injured patients that was not found in the control population [117]. While the decreased connectivity of the hippocampus after chronic injury is unsurprising given findings of significant neuronal cell death within the injured hippocampus [118, 119], the ability to visualize axonal integrity in the patient population further validates the use of DTI as an applicable biomarker discovery tool. Further, DTI can be used to analyze possible biomarkers of injury indicative of cognitive outcome [120, 121]. For example, one study found significantly higher diffusivity in children with TBI correlated with poor social cognitive skills [122]. This study corroborates findings from a 2013 study demonstrating the link between axonal diffusivity and memory in a rodent model of blast injury [123], suggesting a strong case for analyzing white matter abnormalities as not only a marker of injury severity, but one of cognitive dysfunction. A link between motor outcome in injured patients and white matter diffusivity is also being heavily researched, with many studies finding that significantly lower fractional anisotropy (FA) values in patients may be indicators of motor control affect after injury [124, 125]. FA values taken from DTI scans have also shown promise as a predictor of mortality in clinical studies for individuals with severe TBI, demonstrating that DTI is not only useful for mild injury diagnosis [126].

Single photon emission computed tomography (SPECT) is another imaging modality that has high potential for biomarker discovery applications [127]. Approved by the FDA as a diagnosis tool in PD [128, 129], the capability of SPECT to provide true 3D information is beneficial for detection and validation of biomarkers in the patient population. A study conducted by Kinuya et al. in 2004 found that in comparison to CT and MRI analysis, SPECT revealed frontal hypoperfusion and cerebellar hypoperfusion, abnormalities associated with personality change and vertigo respectively [130]. SPECT identifying MRI/CT-negative abnormalities is also seen in both acute and chronic imaging of mild TBI, further demonstrating its utility in the clinic [131]. Furthermore, using ^99m^Tc exametazime in conjunction with SPECT to measure cerebral blood flow (CBF) revealed significantly lower CBF levels in the right temporal lobes of patients with poorer physical health [132]. However promising, candidate biomarkers detected by SPECT appear to lack a strong correlation with cognitive and neuropsychiatric dysfunction, which may affect its clinical utility [132].

## Machine learning and statistical modeling

Machine learning involves using advanced algorithms to analyze large sets of data to progressively recognize patterns without being programmed to do so. Machine learning algorithms can be applied to many categories of datasets, from proteomics to imaging data. This approach is well suited for identifying patterns of disease in biomedical data, and as such, has been applied to biomarker research of many diseases including cancers, psychosis, and Huntington’s disease [133–136]. For biomarker discovery in TBI, machine learning procedures have focused on gathering large amounts of imaging data from the injured patient population. Combining the advancing imaging technology with powerful statistical modeling algorithms has the potential to reveal in depth analysis on prospective biomarkers with direct utility for clinical use, specifically for analyzing white matter connectivity. This approach is evidenced by Mitra et al.’s application of a Network-Based Statistics (NBS) model to fractional anisotropy data [137]. With NBS’s capability of analyzing low contrast-to-noise data, this study revealed sensitivity of 80% when classifying TBI patients [137]. Dynamic functional network connectivity (dFNC) for example is used to analyze global temporal connectivity, but with a linear support vector machine algorithm to classify the data, researchers have found significant connectivity states between cerebellum and sensorimotor networks that may serve as a possible biomarker for classification of mTBI [138]. Similarly, Hellyer et al. applied pattern classification algorithms to DTI data acquired from TBI patients and then applied the classifiers to patients without DTI scans, successfully predicting severity of cognitive impairment induced by injury [139]. Graphical-model-based multivariate analysis (GAMMA), a machine learning tool to analyze interactions between brain regions [140], and tract-based spatial statistics (TBSS) were also be applied to DTI data to use fractional anisotropy values as classifiers to detect neuroimaging biomarkers of mTBI [141]. Additionally, GAMMA has revealed significant differences in the cerebellar white matter integrity between injured and non-injured patients that may have utility as a diagnostic maker of acute stage TBI [142], demonstrating the model’s utility in TBI applications. Predictive algorithms are also utilized with imaging techniques sparsely used for TBI to improve their capability of detecting neurotrauma. In a recent study by Shi et al., a machine learning algorithm was applied to terahertz (THz) continuous-wave (CW) transmission imaging to develop an automatic classification system for diagnosis of TBI [143]. The spatial and temporal power of THz CW imaging proved to be an excellent data source for predictive modeling, with the analysis revealing up to 87.5% classification accuracy [143]. These data demonstrate the capability of machine learning to use or improve upon established imaging techniques to improve accuracy of candidate biomarker discovery.

Machine learning algorithms are versatile in that they can be applied to non-imaging datasets as well. For example, topological data analysis (TDA), a machine learning tool that clusters patient data based on outcome metrics, was used by Nielson et al. to predict novel biomarkers associated with several variables indicative of unfavorable outcome post-injury [144]. The TDA algorithm, which showed great promise in an earlier study involving rodent models of TBI and spinal cord injury [145], analyzed TBI patient data in a multidimensional space, with each patient having over 900 measurable variables. From this model, Nielson et al. found that high levels of specific genetic polymorphisms predicted unfavorable recovery after injury and high probability of PTSD [144]. To analyze and predict protein expression in acute injury, Peacock et al. applied a random forest (RF) predictive model to a panel of biomarkers, including neurogranin, neuron-specific enolase, and metallothionein-3, selected by American Congress of Rehabilitation Medicine criteria [146]. By building a model from this panel, researchers were able to observe the diagnostic accuracy of these biomarkers in predicting mTBI, regardless of neuroimaging findings [146]. RF was also applied to injury data acquired by the American National Football League using metrics including corpus callosum fiber strain and cumulative strain damage of the whole brain to identify predictive concussion biomarkers and evaluate their accuracy [147]. Functional connectivity data detected through magnetoencephalographic recordings can also be analyzed through machine learning methods, revealing that the model was eventually able to discriminate injured patients against controls with 100% accuracy [148]. Interestingly, machine learning algorithms are also incredibly useful for evaluating pediatric TBI cases. When analyzing metrics from physical examination findings, Chong et al.’s application of a machine learning algorithm yielded accuracy above 94% for both sensitivity and specificity [149]. This approach demonstrates the utility of using predicative algorithms for pediatric TBI biomarker discovery and showcases its power in the probability of detecting which biomarkers are indicative of a more aggressive disease progression later in life. Hemodynamics influenced by injury have also been explored as possible biomarkers of TBI, with predictive classification algorithms revealing significant temporal and spatial activity in the prefrontal cortex as possible diagnostic markers of injury [150].

While promising, machine learning algorithms applied to neurotrauma research still have drawbacks. Even though using multivariate analysis is extremely beneficial for analyzing the heterogeneous injury microenvironment, it is critical to consider that larger sample sizes are needed to validate the specificity and sensitivity of the biomarkers selected from these models prior to full utility in clinical applications.

## Conclusion

Several biomarkers of TBI have been identified but they carry the disadvantage of either not being sensitive or specific to TBI, which diminishes their clinical utility. Biomarkers have the potential for improving diagnostic accuracy, predicting the severity of injury progression, and conveying information to clinicians about injury progression for individual patients. Advancements in biomarker discovery range from improving upon already established techniques to applying novel methods to elucidate mechanisms of the neural injury environment. Many emerging tools and techniques have shown promise in inching the field towards a better comprehension of TBI and have given rise to multiple novel candidate biomarkers to further characterize. While preclinical discovery has not yet lead directly to clinical translation, the technological strides discussed here are immensely promising. Ultimately, future efforts in biomarker discovery should continue to rigorously test potential biomarkers and critically inspect their potential clinical utility.

## Abbreviations

AD: Alzheimer’s Disease
ADP: Adenosine diphosphate
BBB: Blood-brain barrier
CBF: Cerebral blood flow
CNS: Central nervous system
CSF: Cerebral spinal fluid
CT: Computerized tomography
CW: Continuous wave
dNFC: Dynamic functional network connectivity
DTI: Diffusion tensor imaging
FA: Fractional anisotropy
GAMMA: Graphical-model-based multivariate analysis
GCS: Glasgow Coma Scale
GFAP: Glial fibrillary acidic protein
miRNA: MicroRNA
MRI: Magnetic resonance imaging
mTBI: Mild TBI
NBS: Network based statistics
NGS: Next generation sequencing
PD: Parkinson’s Disease
SPECT: Single photon emission computed tomography
TAI: Traumatic axonal injury
TBI: Traumatic brain injury
TDA: Topological data analysis
UCH-L1: Ubiquitin carboxyl-terminal hydrolase L1
